# Supplementary material for: End Sequence Analysis Toolkit (ESAT) expands the extractable information from single-cell RNA-seq data
Source: Genome Res. 2016 Oct;26(10):1397–410. doi: 10.1101/gr.207902.116 (PMC5052061; doi:10.1101/gr.207902.116)
Supplement: Supplemental Material [file supp_gr.207902.116_Supp_Materials.tar.gz › ESAT-esat_v0.1/doc/html/classjaligner_1_1formats_1_1_c_l_u_s_t_a_l.html]

ESAT: jaligner.formats.CLUSTAL Class Reference


ESAT


- Main Page
- Related Pages
- Packages
- Classes
- Files

- Class List
- Class Index
- Class Hierarchy
- Class Members

All Classes Namespaces Files Functions Variables Enumerator Pages

- jaligner
- formats
- CLUSTAL

Public Member Functions |
List of all members

jaligner.formats.CLUSTAL Class Reference

Inheritance diagram for jaligner.formats.CLUSTAL:

[legend]

Collaboration diagram for jaligner.formats.CLUSTAL:

[legend]

|  |  |
| --- | --- |
| Public Member Functions | |
|  | CLUSTAL () |
|  | |
| String | format (String[] names, String[] sequences) |
|  | |
| String | format (Alignment alignment) |
|  | |
| Public Member Functions inherited from jaligner.formats.Format | |
| abstract String | format (Alignment alignment) |
|  | |
| void | setId (String id) |
|  | |
| String | getId () |
|  | |

## Detailed Description

CLUSTAL format. Example:

```
CLUSTAL_FORMAT W(1.60) multiple sequence alignment
```

```
JC2395          NVSDVNLNK---YIWRTAEKMK---ICDAKKFARQHKIPESKIDEIEHNSPQDAAE----
KPEL_DROME      MAIRLLPLPVRAQLCAHLDAL-----DVWQQLATAVKLYPDQVEQISSQKQRGRS-----
FASA_MOUSE      NASNLSLSK---YIPRIAEDMT---IQEAKKFARENNIKEGKIDEIMHDSIQDTAE----
```

```
JC2395          -------------------------QKIQLLQCWYQSHGKT--GACQALIQGLRKANRCD
KPEL_DROME      -------------------------ASNEFLNIWGGQYN----HTVQTLFALFKKLKLHN
FASA_MOUSE      -------------------------QKVQLLLCWYQSHGKS--DAYQDLIKGLKKAECRR
```

```
JC2395          IAEEIQAM
KPEL_DROME      AMRLIKDY
FASA_MOUSE      TLDKFQDM
```

Author
:   Ahmed Moustafa (ahmed.nosp@m.@use.nosp@m.rs.sf.nosp@m..net)

## Constructor & Destructor Documentation

|  |  |  |  |  |
| --- | --- | --- | --- | --- |
| jaligner.formats.CLUSTAL.CLUSTAL | ( |  | ) |  |

Constructor

Here is the call graph for this function:

## Member Function Documentation

|  |  |  |  |
| --- | --- | --- | --- |
| String jaligner.formats.CLUSTAL.format | ( | String[] | *names*, |
|  |  | String[] | *sequences* |
|  | ) |  |  |

Returns CLUSTAL format

Parameters
:   |  |  |
    | --- | --- |
    | names | array of the names of the sequences. |
    | sequences | array of the sequences |

Here is the caller graph for this function:

|  |  |  |  |  |  |
| --- | --- | --- | --- | --- | --- |
| String jaligner.formats.CLUSTAL.format | ( | Alignment | *alignment* | ) |  |

Returns CLUSTAL format of the alignment

Parameters
:   |  |  |
    | --- | --- |
    | alignment | (Alignment) |

Returns
:   CLUSTAL format of the alignment

Here is the call graph for this function:

---

The documentation for this class was generated from the following file:

- java/jaligner/formats/CLUSTAL.java


---

Generated on Fri Aug 15 2014 15:11:06 for ESAT by  

 1.8.7
